# Efficacy and safety of anti-PD-1/PD-L1-based dual immunotherapies versus PD-1/PD-L1 inhibitor alone in patients with advanced solid tumor: a systematic review and meta-analysis

**DOI:** 10.1007/s00262-024-03734-1

**Published:** 2024-06-04

**Authors:** Yueying Chen, Hedong Han, Jing Cheng, Qinpei Cheng, Suhua Zhu, Ping Zhan, Hongbing Liu, Yong Song, Tangfeng Lv

**Affiliations:** Department of Respiratory and Critical Care Medicine, Jinling Hospital, Affiliated Hospital of Medical School, Nanjing University, Nanjing, China

**Keywords:** PD-1/PD-L1 inhibitor, Immunotherapy, Therapy combination, Solid tumor

## Abstract

**Introduction:**

Numerous randomized controlled trials (RCTs) have investigated PD-1/PD-L1 inhibitor-based combination therapies. The debate surrounding the potential additive clinical benefits of combination of two immune-oncology (IO) therapies for cancer patients persists.

**Methods:**

Both published and grey sources of randomized clinical trials that compared anti-PD-1/PD-L1-based immunotherapy combinations with monotherapy in patients with advanced or metastatic solid tumors were encompassed. The primary outcome was progression-free survival (PFS), and secondary outcomes included objective response rate (ORR), overall survival (OS) and treatment-related adverse events (TRAEs).

**Results:**

Our analysis encompassed 32 studies comprising 10,341 patients, which covered 12 distinct immune-oncology combination regimens. Across all patients, the immunotherapy combinations exhibited the capability to enhance the ORR (OR = 1.23 [95% CI 1.13–1.34]) and extend PFS (HR = 0.91 [95% CI 0.87–0.95]). However, the observed enhancement in OS (HR = 0.96 [95% CI 0.91–1.01]) was of no significance. Greater benefits in terms of PFS (HR = 0.82 [95% CI 0.72 to 0.93]) and OS (HR = 0.85 [95% CI 0.73 to 0.99]) may be particularly pronounced in cases where PD-L1 expression is negative. Notably, despite a heightened risk of any-grade TRAEs (OR = 1.72 [95% CI 1.40–2.11]) and grade greater than or equal to 3 TRAEs (OR = 2.01 [95% CI 1.67–2.43]), toxicity was generally manageable.

**Conclusions:**

This study suggests that incorporating an additional immunotherapy agent with PD-1/PD-L1 inhibitors can elevate the response rate and reduce the risk of disease progression, all while maintaining manageable toxicity. However, there remains a challenge in translating these primary clinical benefits into extended overall survival.

**Supplementary Information:**

The online version contains supplementary material available at 10.1007/s00262-024-03734-1.

## Introduction

Therapeutic options for patients with inoperable tumors were historically limited to chemotherapy, which often carried significant adverse events and frequent instances of relapse [1]. In the pursuit of enhanced efficacy and improved tolerability, extensive research has brought up novel therapeutic agents and regimens with multifaceted functions, particularly within the realm of cancer immunotherapy which has ushered in a new era of cancer treatment over the past decade [2]. The promising advancements in immune checkpoint inhibitors (ICBs), especially those targeting CTLA-4 or PD-1/PD-L1, have garnered substantial attention due to their favorable clinical outcomes across various malignancies, supported by an array of ongoing trials that are diligently exploring their potential across different indications [3]. However, ICB monotherapy only confers prolonged lifespan to a subset of patients, primarily due to innate and acquired resistance. This limitation underscores the pressing need for alternative strategies that can deliver improved performance and outcomes [4].

The concept of combined immunotherapies targeting multiple immune pathways holds the potential for establishing a durable antitumor immune response and expanding the patient population that could benefit from immunotherapy [5]. Combing PD-1/PD-L1 inhibitor with CTLA-4 inhibitor was demonstrated to amplify antitumor responses synergistically [6–9], and the promising outcomes has led to the approval of the combination therapy for a broad spectrum of malignancies [10].

However, controversies persist over the additional value of IO combinations including synergy of PD-1/PD-L1 inhibitor with another immune checkpoint inhibitor or novel IO drug. Although certain clinical trials have yielded unsatisfactory results with no discernible additional benefit from dual immune checkpoint blockades [11, 12], predefined secondary analyses within the DANUBE study have lent support to the hypothesis that combining durvalumab with tremelimumab might lead to increased activity compared to durvalumab alone. This effect was particularly pronounced in the biomarker-positive population, underscoring the crucial role of precise patient selection in achieving optimal therapeutic benefits [13]. Similarly, clinical trials exploring other immunotherapy combination regimens yielded no certain additional benefit over monotherapy [14, 15]. What’s more, numerous meta-analyses [16–20] consistently indicated a noteworthy rise in the frequency of toxicity incidents when anti-PD-1/PD-L1 and anti-CTLA-4 blockades are administered in tandem.

The discrepancies in clinical efficacy results and concerns regarding toxicity have raised additional doubts about the utilization of IO combinations. Thus, we determined to conduct this meta-analysis aimed at investigating whether the combination of PD-1/PD-L1 blockade with another immunotherapy could offer greater survival benefits to a larger number of patients across various solid tumors while maintaining an acceptable safety profile, as compared to PD-1/PD-L1 blockade alone. The detailed toxicity profiles of adverse events with a potential immunologic cause were also aggregated to provide a clinical reference.

## Materials and methods

This meta-analysis was designed to identify trials employing combination therapies consisting of PD-1/PD-L1-inhibitor-based dual IO therapies that reported efficacy and safety outcomes. It was performed under the guidance of the PRISMA statement [21] and a prospective protocol was registered as INPLASY202370063 (https://inplasy.com).

### Data sources and search strategy

We systematically searched PubMed, EMBASE, Scopus, Cochrane Library databases for relevant trials conducted and reported until November 18, 2023. The ClinicalTrials.gov and abstract books of annual conferences that took place from 2018 to 2023 were also screened in order to include the updated outcomes and unpublished trials, including American Society of Clinical Oncology, European Society of Medical Oncology, Chinese Society of Clinical Oncology, American Association of Cancer Research, and World Conference of Lung Cancer. The keywords for the literature search were “randomized clinical trial, cancer, PD-1, PD-L1, pembrolizumab, atezolizumab, nivolumab, durvalumab, camrelizumab, tislelizumab, sintilimab, toripalimab and serplulimab”.

### Selection criteria

Published and gray sources which met the following criteria were included in this meta-analysis: RCTs enrolling patients with advanced or metastatic solid tumor confirmed either histologically or cytologically; RCTs that used PD-1/PD-L1 inhibitor combining another immunotherapy in either first-line or later treatment settings; RCTs comparing PD-1/PD-L1 inhibitor plus another IO therapy with anti-PD-1/PD-L1 monotherapy for patients with advanced or metastatic solid tumor; phase II or phase III trials reporting at least one of the following clinical outcomes: progression-free survival (PFS), overall survival (OS), objective response rate (ORR), treatment-related Adverse events (TRAEs) of any-grade or grade greater than or equal to 3 TRAEs.

The exclusion criteria were as follows: RCTs that enrolled patients with hematologic malignancies; RCTs comparing dual IO combinations with immunologic monotherapy combined with chemotherapy, tyrosine kinase inhibitor or other treatment modality.

To determine the eligibility, titles and abstracts were screened before assessing full texts. All the included trials were double-checked online to make sure that the most recent data was included.

### Data extraction and quality assessment

Basic information about the trial name, first author, publication year, publication source, National Clinical Trials identification number, cancer type, sample size, patients’ age and sex distribution, names of PD-1 or PD-L1 inhibitor, names of combination agents were extracted. The clinical outcomes we extracted encompassed ORR and hazard ratios (HRs) with corresponding 95% confidence intervals (95% CIs) for PFS and OS. We also recorded the incidence of any-grade TRAEs and grade greater than or equal to 3 TRAEs, along with the safety profile of events experienced by patients who received IO combinations. Part of included trials only provide information about any-grade TRAEs reported in at least 5%, 10% or 15% patients [12, 15, 22–26]. Consequently, any-grade events experienced by ≥ 5% as well as grade ≥ 3 events experienced by ≥ 1% patients in the combination arms were listed after extraction and calculation. Profile of immune-mediated adverse events (imAEs) or adverse events of special interest (AESIs) with a potential immunologic mechanism were also extracted from published reports.

Data extraction was independently done by two investigators (Y.C. and J.C.) and any discrepancies in this process were resolved through discussion with the other investigator (H.H.). The Cochrane Risk of Bias Tool (2.0) for RCTs was applied to check the quality of the included studies.

### Statistical analysis

The primary outcome was PFS, while the secondary outcomes included ORR, OS, any-grade TRAEs, grade greater than or equal to 3 TRAEs and AESIs. HRs with corresponding 95% CIs were utilized to quantify the effect sizes for PFS and OS. On the other hand, ORs with 95% CIs were employed to measure the effect sizes for ORR, any-grade TRAEs, and grade greater than or equal to 3 TRAEs. The risks of each event were measured by risk ratios (RRs) with 95%CIs. Moreover, the statistic inconsistency index (I^2^) was employed to assess the heterogeneity among studies. An I^2^ value exceeding 50% is conventionally considered indicative of substantial heterogeneity, prompting the application of a random-effects model with a logit transformation and subsequent sensitivity analysis. Otherwise, the fixed-effects model was applied. The models were fitted using restricted maximum likelihood estimation, incorporating a classic continuity correction of 0.5 for cells with zero values along with their corresponding sample sizes.

Subgroup analyses were performed based on the type of combination agent, PD-L1 expression status, cancer type and line of treatment. In the subgroup analysis of clinical outcomes for patients with varying PD-L1 expression status, only PFS and OS could be estimated. Given that only two trials reported clinical outcomes for patients with PD-L1 expression exceeding 25%, the patients were categorized into the following groups: less than 1%, greater than or equal to 1%, less than 25%, and greater than or equal to 50%.

Some three-arm trials were initially designed to compare immunotherapy combined with chemotherapy, along with evaluating PD-1/PD-L1 blockade monotherapy against chemotherapy. These trials reported median PFS/OS data and event counts for each group. We used these data to calculate HRs and their corresponding 95% CIs for anti-PD-1/PD-L1-based combinations in comparison to PD-1/PD-L1 monotherapy [27–29]. For studies presenting only Kaplan–Meier curves, the IPDfromKM method [30] was employed to reconstruct individual patient data and conduct survival analysis.

Publication bias was evaluated using the Egger’s regression test in conjunction with a funnel plot. A *p* value of less than 0.10 was deemed significant, suggesting the presence of asymmetry and publication bias. All analytical procedures were conducted using STATA software (version 17) and SPSS statistical software (version 26.0).

## Results

### Systematic review and characteristics of the included studies

**Fig. 1:**
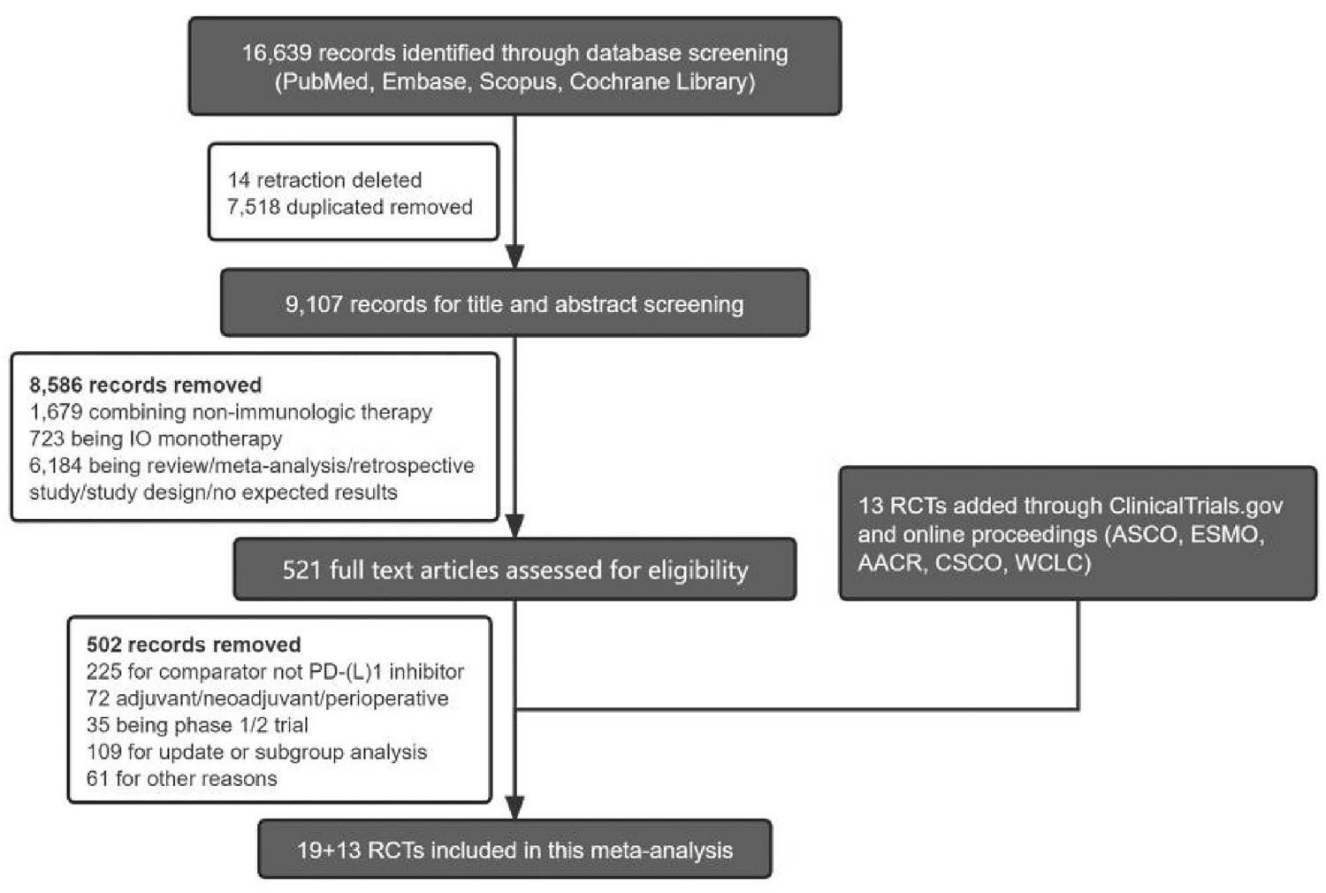
Preferred Reporting Items for Systematic Review and Meta-analyses (PRISMA) diagram

We initially identified a total of 16,639 records across the 4 databases. After removing duplicates and non-pertinent articles through abstract screening, 521 records were deemed eligible for full-text review. Additionally, 13 studies were sourced from ClinicalTrials.gov and conference proceedings. Ultimately, 32 randomized controlled trials were found to meet our eligibility criteria (Fig. 1). Detailed information and baseline characteristics can be found in the provided table (Table 1). Detailed assessment of each randomized controlled trials on the risk of bias is presented in supplementary materials (Supplementary Fig. S1).

**Table 1:** Characteristics of trials included

Trial	Registered ID	Median follow-up duration	Source	Line	Cancer type	Sample Size	Median age	IO combination arm	PD-1/PD-L1 inhibitor
(phase, design)			(year)			(randomization)	(male/female)		
MYSTIC (III, open-label)	NCT02453282	30.2 months	JAMA oncol (2020)	1st	NSCLC	163/163/162 (1:1:1)	64/65/64.5 337/151	Durvalumab + ttremelimumab	Durvalumab
KESTREL (III, open-label)	NCT02551159	4 years	Ann Oncol (2023)	1st	SCCHN	204/413/206 (1:2:1)	62.0/61.0/61.0 (689/134)	Durvalumab + tremelimumab	Durvalumab
EAGLE (III, open-label)	NCT02369874	6.3 months (combo) 7.6 months (mono)	Ann Oncol (2020)	2nd	SCCHN	240/247/249 (1:1:1)	59.0/61.0/61.0 (618/117)	Durvalumab + tremelimumab	Durvalumab
DANUBE (III, open-label)	NCT02516241	41.2 months	Lancet Oncol (2020)	1st	UC	346/342/344 (1:1:1)	67/68/68 (779/253)	Durvalumab + tremelimumab	Durvalumab
ARCTIC-substudy B (III, open-label)	NCT02352948	9.1 months	Ann Oncol (2020)	3rd or later	NSCLC (PD-L1 TC < 25%)	174/118/117/60 (3:2:2:1)	62.5/65.0/63.0/63.5 (308/161)	Durvalumab + tremelimumab	Durvalumab
HIMALAYA (III, open-label)	NCT03298451	46 months (maximum)	ASCO 2022	1st	HCC	393/389/389 (1:1:1)	65.0/64.0/64.0 (987/184)	Durvalumab + tremelimumab	Durvalumab
Lung-MAP S1400I (III, open-label)	NCT02785952	29.5 months	JAMA oncol (2021)	2nd or later	sqNSCLC	125/127 (1:1)	67.5/68.1 (169/83)	Nivolumab + ipilimumab	Nivolumab
CheckMate 227-part1a (III, open-label)	NCT02477826	61.3 months (minimum)	NEJM (2019)	1st	NSCLC (PD-L1 ≥ 1%)	396/396/397 (1:1:1)	64/64/64 (787/402)	Nivolumab + ipilimumab	Nivolumab
CheckMate 067 (III, double-blind)	NCT01844505	77 months	NEJM (2015)	1st	Melanoma	316/314/315 (1:1:1)	59/59/61 (610/335)	Nivolumab + ipilimumab	Nivolumab
KEYNOTE-598 (III, double-blind)	NCT03302234	20.6 months	JCO (2021)	1st	NSCLC (PD-L1 TPS ≥ 50%)	284/284 (1:1)	64/65 (393/175)	Pembrolizumab + ipilimumab	Pembrolizumab
MASTERKEY-265 (III, double-blind)	NCT02263508	31.0 months	JCO (2023)	1st or 2nd	Melanoma	346/364 (1:1)	64/64 (418/274)	Pembrolizumab + T-VEC	Pembrolizumab
PIVOT IO 001 (III, open-label)	NCT03635983	11.6 months	JAMA oncol (2020)	1st	Melanoma	391/392 (1:1)	61.3/60.4 (458/325)	Nivolumab + bempegaldesleukin	Nivolumab
ECHO-301/KEYNOTE-252 (III, double-blind)	NCT02752074	12.4 months	Lancet Oncol (2019)	1st	Melanoma	354/352 (1:1)	64/63 (423/283)	Pembrolizumab + epacadostat	Pembrolizumab
ECHO-303/KEYNOTE-698 (III, open-label)	NCT03374488	Time Frame: 27 months	ClinicalTrials.gov	2nd	UC	42/42 (1:1)	67.9/65.2 (72/12)	Pembrolizumab + epacadostat	Pembrolizumab
ECHO-304/KEYNOTE-669 (III, open-label)	NCT03358472	Time Frame: 14 months	ClinicalTrials.gov	1st	SCCHN	35/19/35 (2:1:2)	62.1/63.0/62.7 (75/14)	Pembrolizumab + epacadostat	Pembrolizumab
ECHO-307/KEYNOTE-672 (III, open-label)	NCT03361865	Time Frame: 25 months	ClinicalTrials.gov	1st	UC	44/49 (1:1)	73.3/72.4 (71/22)	Pembrolizumab + epacadostat	Pembrolizumab
CA017-055 (III, open-label)	NCT03329846	Time Frame: 25 months	ClinicalTrials.gov	1st	Melanoma	10/10 (1:1)	– (12/8)	Nivolumab + BMS-986205	Nivolumab
RELATIVITY-047 (II-III, double-blind)	NCT03470922	25.3 months	NEJM (2022)	1st	Melanoma	355/359 (1:1)	63.0/62.0 (416/298)	Nivolumab + relatimab	Nivolumab
INDUCE-3 (II-III,double-blind)	NCT04128696	Time Frame: 16 months	ClinicalTrials.gov	1st	SCCHN (PD-L1 CPS ≥ 1)	158/157 (1:1)	− 255/60	Pembrolizumab + feladilimab	Pembrolizumab
CONDOR (II,open-label)	NCT02319044	6.5 months (combo) 6.0 months (mono)	JAMA oncol (2018)	2nd	SCCHN (PD-L1 TC < 25%)	133/67/67 (2:1:1)	62/62/61 (220/47)	Durvalumab + tremelimumab	Durvalumab
CCTG IND 232 (II,open-label)	NCT02788773	Time frame: 2 years	ESMO 2019	2nd or later	CRPC	39/13 (3:1)	–	Durvalumab + tremelimumab	Durvalumab
CALYPSO (II,open-label)	NCT02819596	Time frame: 18 months	ASCO 2022	2nd	RCC	39/39 (1:1)	–	Durvalumab + tremelimumab	Durvalumab
NRG GY003 (II, open-label)	NCT02498600	33.8 months (combo) 33.4 months (mono)	JCO (2020)	2nd to 4th	Ovarian cancer	51/49 (1:1)	62/63 (/)	Nivolumab + ipilimumab	Nivolumab
CheckMate 714 (II, double-blind)	NCT02823574	22.7 months (minimum)	JAMA oncol (2023)	1st (platinum-refractory)	SCCHN	159/82 (2:1)	59.0/58.0 (194/47)	Nivolumab + ipilimumab	Nivolumab
		22.2 months (minimum)		1st(platinum-eligible)		123/61 (2:1)	61.0/62.0 (152/32)		
EMPOWER-Lung 4 (II, open-label)	NCT03430063	Time Frame: 41 months	ESMO 2020	2nd	NSCLC	11/8 (1:1)	68.5/62.4 (13/6)	Cemiplimab + ipilimumab	Cemiplimab
CITYSCAPE (II, double-blind)	NCT03563716	30.4 months	Lancet Oncol (2022)	1st	NSCLC (PD-L1 TPS ≥ 1%)	67/68 (1:1)	68/68 (87/48)	Atezolizumab + tiragolumab	Atezolizumab
ECHO-305/KEYNOTE-654 (II, double-blind)	NCT03322540	Time Frame: 36 months	ClinicalTrials.gov	1st	NSCLC (PD-L1 TPS ≥ 50%)	77/77 (1:1)	63.7/66.9 (112/42)	Pembrolizumab + epacadostat	Pembrolizumab
CA017-056 (II, open-label)	NCT04106414	Time Frame: 1 year	ASCO 2022	2nd to 5th	Endometrial Cancer	12/12 (1:1)	–	Nivolumab + BMS-986205	Nivolumab
NCT03513952 (II, open-label)	NCT03513952	Time Frame: 2 years	ESMO 2023	2nd or later	UC	26/21 (1:1)	–	Atezolizumab + CYT107	Atezolizumab
CYPRESS 1 (II, open-label)	NCT03382899	10.0 months	JTO (2020)	1st	NSCLC (PD-L1 TPS ≥ 50%)	51/50 (1:1)	69/68 (60/41)	Pembrolizumab + pegilodecakin	Pembrolizumab
CYPRESS 2 (II, open-label)	NCT03382912	11.6 months	JTO (2020)	2nd or later	NSCLC (PD-L1 TPS < 50%)	27/25 (1:1)	70/66 31/21	Nivolumab + pegilodecakin	Nivolumab
NCT02609984 (II,open-label)	NCT02609984	18.1 months (combo) 17.7 months (mono)	JCO (2021)	2nd or later	Sarcoma (NY-ESO-1 positive)	45/44 (1:1)	46.2/46.7 (51/38)	Atezolizumab + CMB305	Atezolizumab

For trials with no median follow-up duration reported in publications, the expected time frame given on ClinicalTrials.gov is listed for reference.

Definitions of abbreviations: NSCLC, non-small-cell lung cancer; SCCHN, squamous-cell cancer of head and neck; UC, urothelial cancer; HCC, hepatocellular carcinoma; sqNSCLC, non-squamous non-small-cell lung cancer; CRPC, castrarion-resistant prostate cancer; RCC, renal cell cancer; TPS, tumor cell proportion score; TC, tumor cell; CPS, combined positive score; combo, IO combination therapy; mono, PD-1/PD-L1 inhibitor monotherapy

### Comparison of PFS, OS and ORR

**Fig. 2:**
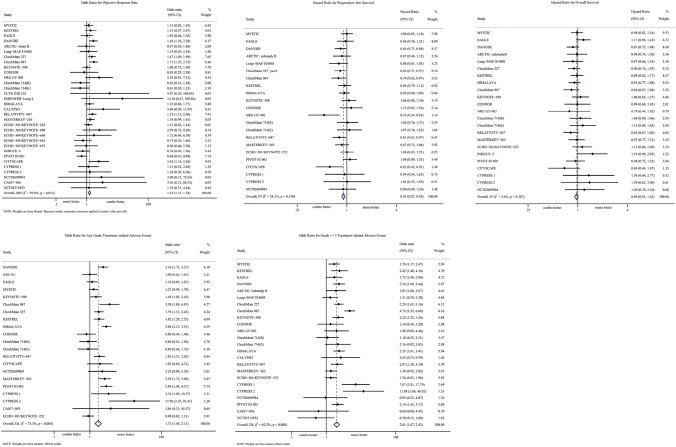
Forest plots of ORR, PFS, OS, any-grade TRAEs and grade ≧ 3 TRAEs for anti-PD-1/PD-L1-based immunotherapy combinations compared with PD-1/PD-L1 blockade monotherapy. Combo:combination therapy; mono: m onotherapy

21 studies included in this meta-analysis provide information about PFS. Generally, patients receiving PD-1/PD-L1-inhibitor-based IO combinations were prone to longer survival without progression (HR = 0.91 [95% CI 0.87–0.95]). Among the 32 trials included in this meta-analysis, 31 reported ORR, with some of them additionally providing information for comparing OS. In general, combination therapies based on PD-1/PD-L1 blockade exhibited a higher response rate (OR = 1.24 [95% CI 1.13–1.35]). When considering OS, IO combinations conferred greater benefit compared to PD-1 or PD-L1 inhibitor monotherapy (HR = 0.96 [95% CI 0.91–1.01]), although the observed differences in OS were not statistically significant (Fig. 2).

### Comparisons of any-grade TRAEs and grade ≥ 3 TRAEs

Safety and toxicity were evaluated based on the incidence of any-grade TRAEs and grade greater than or equal to 3 TRAEs. Specifically, 16 and 22 studies were respectively incorporated to assess any-grade TRAEs and grade greater than or equal to 3 TRAEs.

The combination of two immunotherapeutic agents exhibited a significant increase in toxicity (OR = 1.72 [95% CI 1.40–2.11]), particularly in relation to the elevated risk of higher-grade adverse events (OR = 2.01 [95% CI 1.67–2.43]) (Fig. 2). Among the total of 17 clinical trials that provided detailed information about any-grade TRAEs in their articles or supplementary materials, the most frequently reported adverse events for IO combinations encompassed fatigue, chills, pruritus, diarrhea, and pyrexia. Notably, special attention is warranted for infusion-related rash (RR = 17.78 [95% CI 1.05–300.08]), chills (RR = 6.31 [95% CI 3.49–11.42]) and pyrexia (RR = 4.08 [95% CI 3.03–5.48]), all of which displayed significantly elevated risk ratios. When considering grade greater than or equal to 3 TRAEs, clinicians are advised to exercise vigilance regarding anemia (RR = 4.10 [95% CI 1.17–14.34]), thrombocytopenia (RR = 4.73 [95% CI 2.32–9.65]), diarrhea (RR = 3.37 [95% CI 2.13–5.34]), and colitis (RR = 3.93 [95% CI 1.92–8.08]) (Supplementary Table 2).

Among adverse events of special interest (AESIs), a heightened focus may be required on sarcoidosis (RR = 8.96 [95% CI 0.48–166.13]), hypopituitarism (RR = 6.04 [95% CI 0.73–49.99]), increases in ALT (RR = 4.58 [95% CI 2.50–8.39]) or AST (RR = 4.00 [95% CI 2.17–7.38]), autoimmune hepatitis (RR = 3.99 [95% CI 0.45–35.51]), and colitis (RR = 3.78 [95% CI 2.47–5.80]). Notably, greater attention should be directed towards grade ≥ 3 adverse events with a potential inflammatory or immunologic cause, including pruritus (RR = 17.07 [95% CI 0.99–295.31]), hypopituitarism (RR = 9.05 [95% CI 0.491–67.81]), increases in ALT (RR = 6.50 [95% CI 2.30–18.41]) or AST (RR = 6.33 [95% CI 1.89–21.19]), and type 1 diabetes mellitus (RR = 5.51 [95% CI 0.70–45.75]) (Supplementary Table 3).

### Subgroup analysis

#### On the basis of combination agent

Given the approved usage of dual immune checkpoint inhibitors targeting PD-1/PD-L1 and CTLA-4 across a range of malignancies, we categorized the included trials into two distinct groups: “anti-PD-1/PD-L1 plus anti-CTLA-4” and “anti-PD-1/PD-L1 plus another IO therapy.” In comparison to PD-1/PD-L1 inhibitor monotherapy, the addition of a CTLA-4 inhibitor demonstrated an increase in the response rate (OR = 1.29 [95% CI 1.15–1.44]) and potential extension of survival without progression (HR = 0.91 [95% CI 0.86–0.96]). A subtle variation in OS was also observed (HR = 0.96 [95% CI 0.90–1.02]). Correspondingly, the combined therapy exhibited an elevated incidence of adverse events. The odds ratio was 1.52 [95% CI 1.19–1.94] for any-grade TRAEs and 2.11 [95% CI 1.74–2.57] for grade greater than or equal to 3 TRAEs (Supplementary Fig. S2).

Furthermore, the combination of anti-PD-1/PD-L1 with a different type of immunotherapy demonstrated enhancements in the ORR (OR = 1.15 [95% CI 1.01–1.32]) and PFS (HR = 0.91 [95% CI 0.83–1.00]). However, these novel combinatorial regimens were comparatively less effective than the traditional pairing of PD-1/PD-L1 inhibitor and CTLA-4 inhibitor. Notably, there were minimal differences between combination therapy and monotherapy concerning OS (HR = 0.98 [95% CI 0.88–1.09]). The risk of any-grade (OR = 2.25 [95% CI 1.50–3.38]) and grade equal–or higher than 3 TRAEs (OR = 1.88 [95% CI 1.25–2.83]) were both significantly higher than PD-1/PD-L1 blockade alone (Supplementary Fig. S2).

#### On the basis of PD-L1 status

The most pronounced trend toward enhanced PFS (HR = 0.82 [95% CI 0.72–0.93]) was evident among patients with a lower PD-L1 expression (less than 1%) accompanied by improved OS (HR = 0.85 [95% CI 0.73–0.99]). Among patients with PD-L1 expression greater than or equal to 1%, the combination therapy were capable of prolonging survival without progression (HR = 0.95 [95% CI 0.87–1.04]). However, when compared to PD-1/PD-L1 inhibitor monotherapy, the addition of another immunotherapy did not yield notable benefits in terms of OS (HR = 1.00 [95% CI 0.90–1.11]) (Supplementary Fig. S3).

Regarding patients with PD-L1 expression less than 25%, the results were comparable, demonstrating an improvement in PFS (HR = 0.95 [95% CI 0.84–1.09]) and minimal deviation in OS (HR = 0.99 [95% CI 0.86–1.14]). For patients with PD-L1 expression greater than or equal to 50%, the combination of PD-1/PD-L1 blockade and another immunotherapy also demonstrated a potential reduction in the risk of disease progression (HR = 0.90 [95% CI 0.80–1.02]) and death (HR = 0.99 [95% CI 0.87–1.13]). These results implied a potential link between PD-L1 status and efficacy (Supplementary Fig. S3).

#### On the basis of *cancer* type

To evaluate clinical efficacy and safety based on cancer type, patients were categorized into four groups: non-small-cell lung cancer (NSCLC), head and neck squamous cell cancer (HNSCC), melanoma, and other.

For patients with NSCLC, the combination of two IO therapies demonstrated improvements in both PFS (HR = 0.90 [95% CI 0.83–0.98]) and OS (HR = 0.95 [95% CI 0.87–1.04]), as deduced from data extracted from eight trials analyzed in the subgroup analysis. Patients with melanoma exhibited a comparable trend, with extended PFS (HR = 0.90 [95% CI 0.83–0.98]) and OS (HR = 0.92 [95% CI 0.82–1.02]). However, the outcomes for patients with HNSCC were less encouraging, indicating a potential lack of benefit in terms of PFS (HR = 0.99 [95% CI 0.89–1.11]) and OS (HR = 1.09 [95% CI 0.98–1.21]). Conversely, among patients with other types of cancer, there appeared to be a lower risk of disease progression (HR = 0.87 [95% CI 0.78–0.96]) as well as the inspiriting improvement in OS (HR = 0.90 [95% CI 0.80–1.01]) (Supplementary Fig. S4).

Turning to odds ratios for any-grade TRAEs, other types of cancer displayed the highest incidence (OR = 2.60 [95% CI 2.09–3.23]), followed by melanoma (OR = 2.12 [95% CI 1.34–3.35]) and NSCLC (OR = 1.63 [95% CI 1.19–2.24]). Notably, patients with NSCLC were more prone to experiencing grade greater than or equal to 3 TRAEs (OR = 2.31 [95% CI 1.64–3.26]). Pooling available data, the collective outcomes indicated that combination immunologic therapies were generally well-tolerated in patients with HNSCC, as evidenced by odds ratios for any-grade and grade greater than or equal to 3 TRAEs of 1.13 [95% CI 0.82– 1.56] and 1.77 [95% CI 1.32–2.39], respectively (Supplementary Fig. S4).

#### On the basis of treatment setting

Immunotherapy combination seems to be more advantageous over PD-1/PD-L1 inhibitor alone to improve response rate in the post-line treatment setting (OR = 1.33 [95%CI 1.09–1.62]), but is more beneficial to prolong overall survival in the first-line treatment (OR = 0.95 [95%CI 0.89–1.01]). The increased incidence of adverse events while receiving IO combination regimens was more pronounced in first-line application than in non-first-line application. (Supplementary Fig. S5).

### Heterogeneity analysis and publication *bias*

To validate the consistency of our findings, sensitivity analyses were performed by systematically omitting one study at a time to evaluate if the aggregated outcomes were significantly influenced by any particular trial. Encouragingly, the results indicated that the overall estimates remained stable throughout these evaluations. A marginal to moderate level of heterogeneity across the included trials was identified. Meta-regression analysis was conducted to delve into the potential sources of heterogeneity in the pooled outcomes of any-grade and grade higher than or equal to 3 TRAEs. Trial phase, line of treatment, cancer type, type of PD-1/PD-L1 inhibitor, and target of the combination agent were considered as categorial coefficients while the sources of heterogeneity remained challenging to fully elucidate and explain (Supplementary Table 4). No significant evidence of publication bias was observed, as the funnel plots displayed no discernible asymmetry (Supplementary Fig. S6). This observation was further confirmed through the application of Egger’s test (*p* = 0.673) (Supplementary Table 5).

## Discussion

Immunotherapy has changed the landscape of cancer treatment, especially the encouraging performance of ICBs, and combination therapies arose with the aim of restoring immune function. However, whether the combination could outperform monotherapy remains controversial. To our knowledge, this meta-analysis is the first to directly compare clinical efficacy and safety of PD-1/PD-L1 inhibitor-based IO combinations with single PD-1/PD-L1 inhibitor. The pooled results indicate that combining anti-PD-1/PD-L1 with another immunotherapeutic agent is able to augment both response rates and progression-free survival, all while maintaining a manageable level of treatment-related adverse events which can be resolved with the help of therapeutic agents.

Combination therapies may bolster antitumor immune response and broaden patient population who might benefit from immunotherapy [5]. In contrast to CTLA-4, which operates within lymph nodes to dampen T-cell activation, PD-1 plays a role in hindering T effector cells and promoting Treg differentiation in peripheral tissues [1]. The spatial segregation of functions, along with their complementary and non-redundant roles in effectively targeting both CD8^+^T cells and CD4 + T cells, underscore the rationale for combination therapy [31]. Other PD-1/PD-L1 inhibitor-based strategies have also been hypothesized to synergistically enhancing T-cell responses and overcoming PD-L1-related immune evasion within the intricate tumor microenvironment [14, 32]. Beyond the realm of immune checkpoint molecules, targeting co-stimulatory pathways play a pivotal role in bolstering T cell activity [33] and revitalizing antitumor immune responses [34]. Moreover, a spectrum of immune and non-immune cell types, along with the myriad factors they secrete, has been identified within the tumor microenvironment (TME). These elements collectively establish a chronic inflammatory, immunosuppressive, and pro-angiogenic intertumoral milieu [35]. Cytokines orchestrating critical interactions between immune and non-immune cells, such as IL-2, IL-12, and IL-15, have been harnessed [36] and demonstrated the potential to rejuvenate exhausted T cells [37], decrease regulatory T cells (Treg) in the TME, or reprogram macrophage polarization [36], thus enhancing tumor control in synergy with immune checkpoint inhibitors in preclinical models and early-phase clinical trials [38–41]. A series of clinical investigations involving novel immunotherapeutic agents are underway [42–45]. Furthermore, preclinical investigations and early-phase clinical trials have demonstrated enhanced anti-tumor efficacy and safety of bispecific antibodies compared to traditional multi-target therapies, including combination approaches [37, 46–51]. Notably, the improvement in OS was of no statistical significance. This may be explained by acquired drug resistance, and the mechanisms behind include acquisition of mutations in essential pathways of immunotherapy response, the loss of tumor neoantigens, co-inhibition molecules in the tumor microenvironment and so on [52]. Given the complicated and dynamic TME, further studies exploring how to translate benefit in tumor response and progression-free survival into prolonged overall survival are needed.

The intriguing observations in our study provoke inquiries into the reliability of PD-L1 as a dependable biomarker for patient selection at low cutoffs and within the realm of combination therapies. An interesting observation emerged when examining the PD-L1 subgroups. Specifically, the group with PD-L1 expression < 1% exhibited a more favorable response to combination therapy compared with monotherapy, as opposed to the PD-L1 ≥ 1% group. This intriguing phenomenon could potentially be attributed to the distinctive immune effects of anti-PD-1 and anti-CTLA-4 agents, which might hold particular significance in PD-L1-negative tumors [23]. Thus, the expression level of PD-L1 may not accurately represent the PD-1/PD-L1 interaction state and anti-tumor immunity [53]. The reduced reliance on PD-L1 expression in the context of IO combinations opened avenues for in-depth exploration into the intricate mechanisms at play and paved the way for the identification of more dependable biomarkers guiding patient selection. Notably, tumor mutational burden (TMB) was proposed to serve as a more robust biomarker independent of PD-L1 expression level for immunotherapy combinations [54]. There is growing recognition of other conceivable biomarkers encompassing microsatellite instability [55] as well as signatures closely tied to tumor inflammation, including those derived from analyses of inflammatory gene expression profiles, which hold promise as additional potential indicators [56]. Given the inherent tumor heterogeneity, the creation of a comprehensive framework that amalgamates PD-L1 expression status, tumor mutational burden (TMB), personalized immune profiling, genomic, transcriptomic, and microbiome data could substantially enhance predictive accuracy and facilitate future interventions [4, 10].

The most frequently reported adverse events for IO combinations in our study encompassed fatigue, chills, pruritus, diarrhea, and pyrexia, generally in line with previous study focused on treatment-related adverse events [3]. Special attention is warranted for infusion-related rash, chills and pyrexia, all of which displayed significantly elevated risk ratios. Notably, the occurrence of infusion-related rash was solely documented in 2 trials [57], possibly attributable to pegilodecakin. Given that profiles of imAEs or AESI with a potential immunologic mechanism was available in only 10 studies, we aggregated and analyzed this data to provide a comprehensive overview of events warranting particular attention. Lung cancer displayed a lower risk of all-grade and grade 3 or higher TRAEs compared to melanoma and urinary system tumors in the prior investigation [3], which was contrary to our results. This discrepancy may be attributed to the limited sample size of each cancer type in our study to some extent. What’s more, it was speculated that the prior history of chemotherapy might induce a lower baseline immunoactivity and potentially shield patients from immune system over activation. This is due to the immunosuppressive effect exhibited by chemotherapy agents, arising from their anti-proliferative impact on immune cells [58]. The influence of pretreatment may also explain the discrepancies observed to some degree, highlighting the need for further exploration and validation through additional studies. Subgroup analyses of melanoma patients revealed significant heterogeneity in clinical efficacy and safety compared to other cancer types. The heterogeneity observed may arise from the diverse baseline patient characteristics and therapeutic agents with distinct target molecules and mechanisms of action.

There are several limitations to this study. Firstly, limited by number of clinical trials directly comparing IO combinations with PD-1/PD-L1 inhibitor alone, the trials included were diverse so that the heterogeneity was hard to be fully elucidated. Not enough follow-up duration of some trials led to the limited dataset for PFS and OS statistics which could potentially constrain comprehensive investigations as well as adding to the heterogeneity of pooled results. Consequently, future randomized controlled trials are essential to suffice the pooled outcomes and more efforts should be made to explore efficient treatment combinations. Secondly, while the included studies encompassed various PD-1/PD-L1 inhibitor-based combination regimens, not all of them reported outcomes for patients with different PD-L1 expression statuses, which may hinder further analyses based on PD-L1 expression levels. Thirdly, it is important to note that some of the studies included were not explicitly designed for a formal statistical comparison between the two groups: single PD-1/PD-L1 inhibitor and PD-1/PD-L1 inhibitor-based IO combinations, calling for secondary data processing. Furthermore, future studies focusing on specific cancer types are imperative. This is particularly important considering the distinct immunogenicity profiles exhibited by different malignancies [59].

In conclusion, safety and toxicity assessments unveiled that the addition of another I-O therapy led to increased incidence of treatment-related adverse events as well as adverse events with a potential immunologic mechanism. Nevertheless, the toxicity profiles were generally manageable. Clinical efficacy was predominantly gauged by progression-free survival, overall survival, and objective response rate. The introduction of an anti-CTLA-4 inhibitor or other I-O therapy was associated with heightened objective response rates, translating to reduced risks of disease progression and mortality. The incorporation of combination therapies may also confer improvements in overall survival, although the difference was slight and statistical significance for this outcome was not achieved. Notably, the augmentation of clinical efficacy appears more pronounced in patients exhibiting high PD-L1 expression levels. Consequently, a pressing unmet need persists for identifying an optimal treatment combination that extends the lifespan of individuals with advanced or metastatic cancer, while minimizing the burden of heightened drug-related toxic effects.

## Supplementary Information

Below is the link to the electronic supplementary material.

Supplementary file1 (DOCX 9354 KB)
