# Supplementary material for: Efficacy and safety of anti-PD-1/PD-L1-based dual immunotherapies versus PD-1/PD-L1 inhibitor alone in patients with advanced solid tumor: a systematic review and meta-analysis
Source: Cancer Immunol Immunother. 2024 Jun 4;73(8):155. doi: 10.1007/s00262-024-03734-1 (PMC11150353; doi:10.1007/s00262-024-03734-1)

**Supplementary Table 1**. Literature search strategy.

| Database | Keywords |
| --- | --- |
| PubMed |  |
| #1 | ((((((((((((immune checkpoint inhibitor[Title/Abstract]) OR (immune checkpoint blockade[Title/Abstract])) OR (PD-1[Title/Abstract])) OR (PD-L1[Title/Abstract])) OR (nivolumab[Title/Abstract])) OR (pembrolizumab[Title/Abstract])) OR (durvalumab[Title/Abstract])) OR (atezolizumab[Title/Abstract])) OR (sintilimab[Title/Abstract])) OR (tislelizumab[Title/Abstract])) OR (camrelizumab[Title/Abstract])) OR (toripalimab[Title/Abstract])) OR (serplulimab[Title/Abstract]) |
| #2 | (("solid tumor"[Title/Abstract]) OR (tumor[Title/Abstract])) OR (cancer[Title/Abstract]) |
| #3 | ((("randomized controlled trial"[Title/Abstract]) OR (randomized[Title/Abstract])) OR (randomization[Title/Abstract])) OR (randomly[Title/Abstract]) |
| #4 | #1 AND #2 AND #3 |
| Scopus | (TITLE-ABS-KEY ("randomized controlled trial" OR randomized OR randomization OR randomly) AND TITLE-ABS-KEY ( cancer OR tumor OR “solid tumor”) AND TITLE ( "immune checkpoint inhibitor" OR "immune checkpoint blockade" OR PD-1 OR PD-L1 OR nivolumab OR pembrolizumab OR durvalumab OR atezolizumab OR sintilimab OR tislelizumab OR camrelizumab OR toripalimab OR serplulimab) ) AND ( LIMIT-TO ( DOCTYPE , "ar" ) ) |
| Embase |  |
| #1 | (‘solid tumor’ OR tumor OR cancer):ti,ab,kw |
| #2 | (‘immune checkpoint inhibitor’ OR ‘immune checkpoint blockade’ OR PD-1 OR PD-L1 OR nivolumab OR pembrolizumab OR durvalumab OR atezolizumab OR sintilimab OR tislelizumab OR camrelizumab OR toripalimab OR serplulimab): ti,ab,kw |
| #3 | #1 AND #2 AND [randomized controlled trial]/lim |
| Cochrane |  |
|  | Title Abstract Keyword: ‘cancer’ OR ‘tumor’ OR ‘solid tumor’ |
| AND | Title Abstract Keyword: ‘immune checkpoint inhibitor’ OR ‘immune checkpoint blockade’ OR PD-1 OR PD-L1 OR nivolumab OR pembrolizumab OR durvalumab OR atezolizumab OR sintilimab OR tislelizumab OR camrelizumab OR toripalimab OR serplulimab |
| AND | Title Abstract Keyword: ‘randomized controlled trial’ |

**Supplementary Table 2**. Spectrum of adverse events related to treatment.

**Supplementary Table 3**. Spectrum of adverse events of special interest.

**Supplementary Table 4**. Result of meta-regression analysis

| Meta-regression Anlaysis of Any-grade TRAEs | | | | | |
| --- | --- | --- | --- | --- | --- |
| Covariates | Phase | Line of Treatment | Cancer Type | PD-1/PD-L1 Inhibitor | Combination Therapy |
| Coef. | 0.192 | 0.118 | 0.932 | 0.719 | 0.8 |
| Std.Err. | 0.282 | 0.279 | 0.261 | 0.483 | 0.648 |
| p > \|t\| | 0.505 | 0.676 | 0.002 | 0.154 | 0.239 |
| tau² | 0.164 | 0.176 | 0.112 | 0.209 | 0.16 |
| I² | 74.98% | 75.69% | 67.39% | 78.09% | 74.89% |

| Meta-regression Anlaysis of Grade ≥ 3 TRAEs | | | | | |
| --- | --- | --- | --- | --- | --- |
| Covariates | Phase | Line of Treatment | Cancer Type | PD-1/PD-L1 Inhibitor | Combination Therapy |
| Coef. | -0.051 | 0.304 | 0.541 | -0.05 | 0.309 |
| Std.Err. | 0.262 | 0.202 | 0.237 | 0.889 | 0.333 |
| p > \|t\| | 0.849 | 0.147 | 0.034 | 0.956 | 0.367 |
| tau² | 0.118 | 0.074 | 0.125 | 0.102 | 0.071 |
| I² | 65.82% | 57.36% | 65.83% | 62.12% | 49.42% |

**Supplementary Table 5**. Publication bias tests by funnel plots and egger’ s tests of ORR, PFS, OS, any-grade TRAEs and grade ≥ 3 TRAEs.

|  | ORR | PFS | OS | Any-grade TRAEs | Grade ≥ 3 TRAEs |
| --- | --- | --- | --- | --- | --- |
| Coefficient | 0.234 | -0.318 | 0.908 | 0.513 | -0.248 |
| Std.err | 0.448 | 0.742 | 0.557 | 1.144 | 0.860 |
| t | 0.52 | -0.43 | 1.63 | 0.45 | -0.29 |
| P>\|t\| | 0.605 | 0.673 | 0.118 | 0.659 | 0.775 |

**Supplementary Fig. S1**. Summary of quality assessments using Cochrane Risk of Bias Tool 2.0.


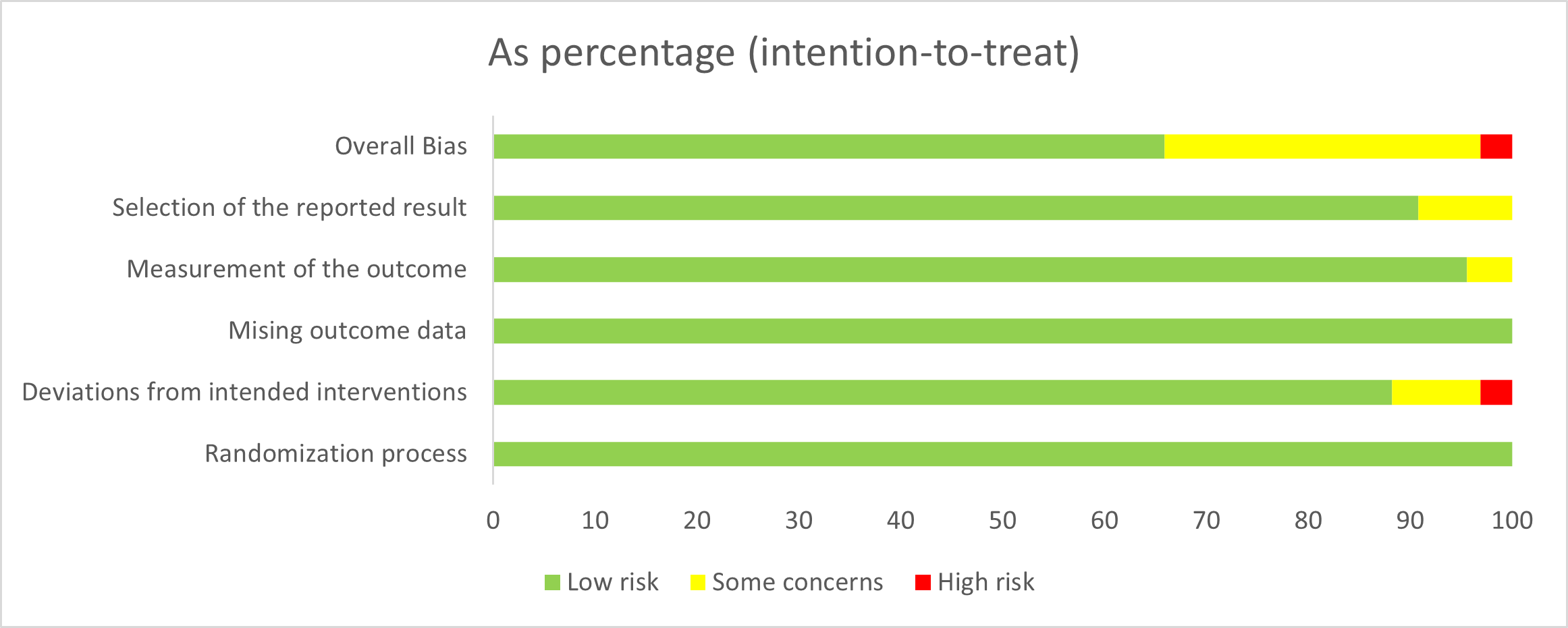


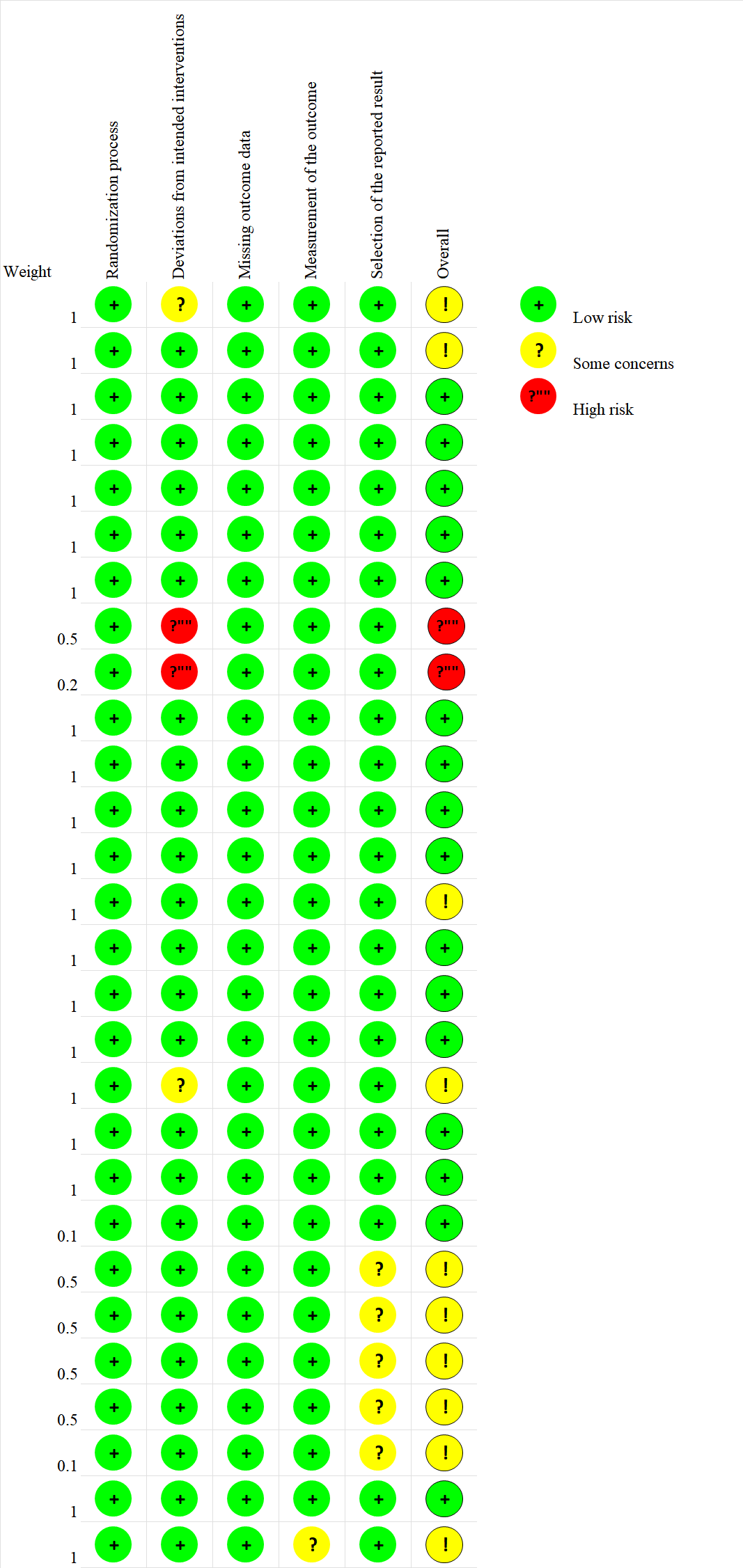


**Supplementary Fig. S2** Forest plots of subgroup analyses of ORR, PFS, OS, any-grade TRAEs and grade higher than or equal to 3 TRAEs based on the type of combination immunotherapeutic agent. Combo: combination therapy; Mono: monotherapy.


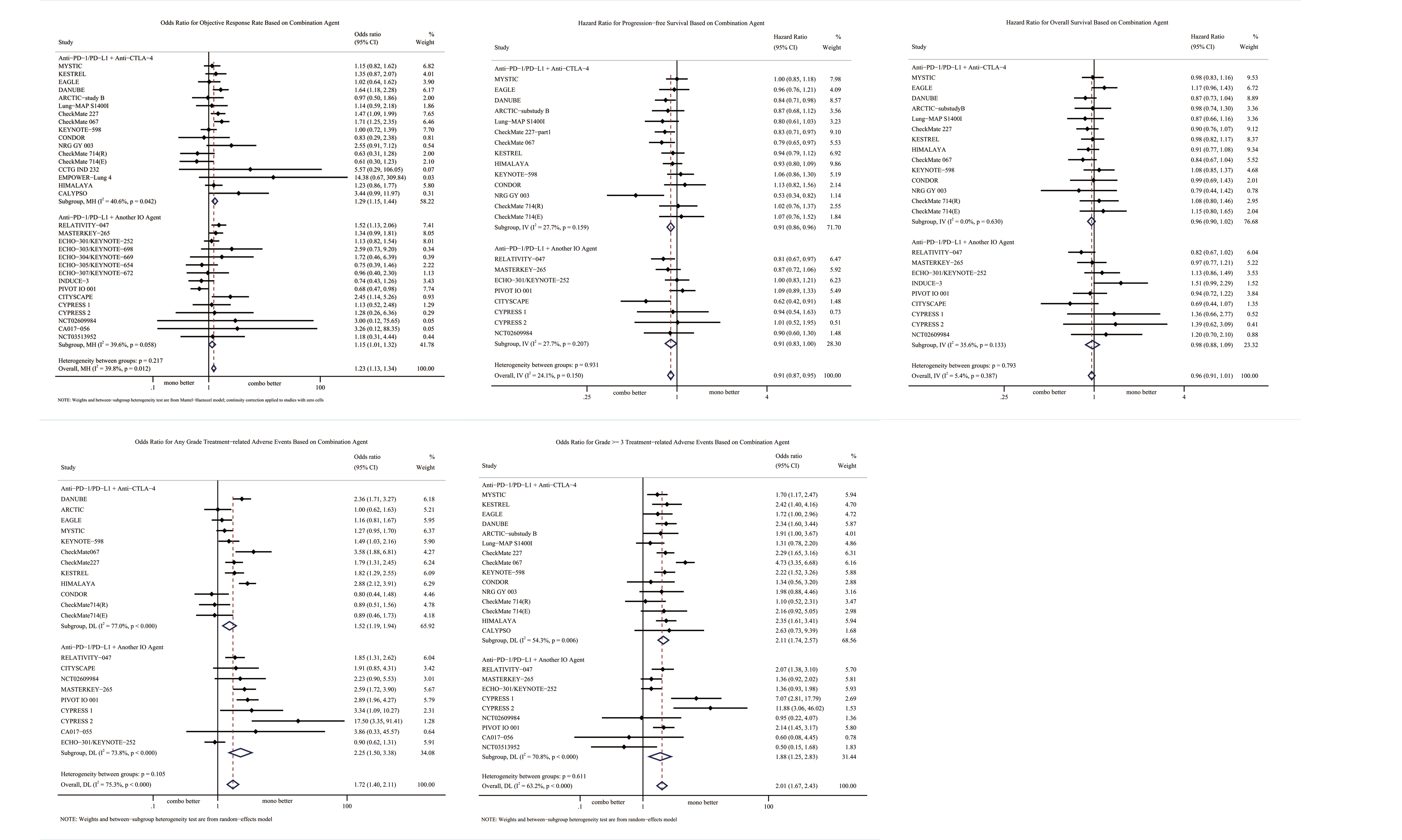


**Supplementary Fig. S3** Forest plots of subgroup analyses of PFS and OS based on PD-L1 expression status. Combo: combination therapy; mono: monotherapy.


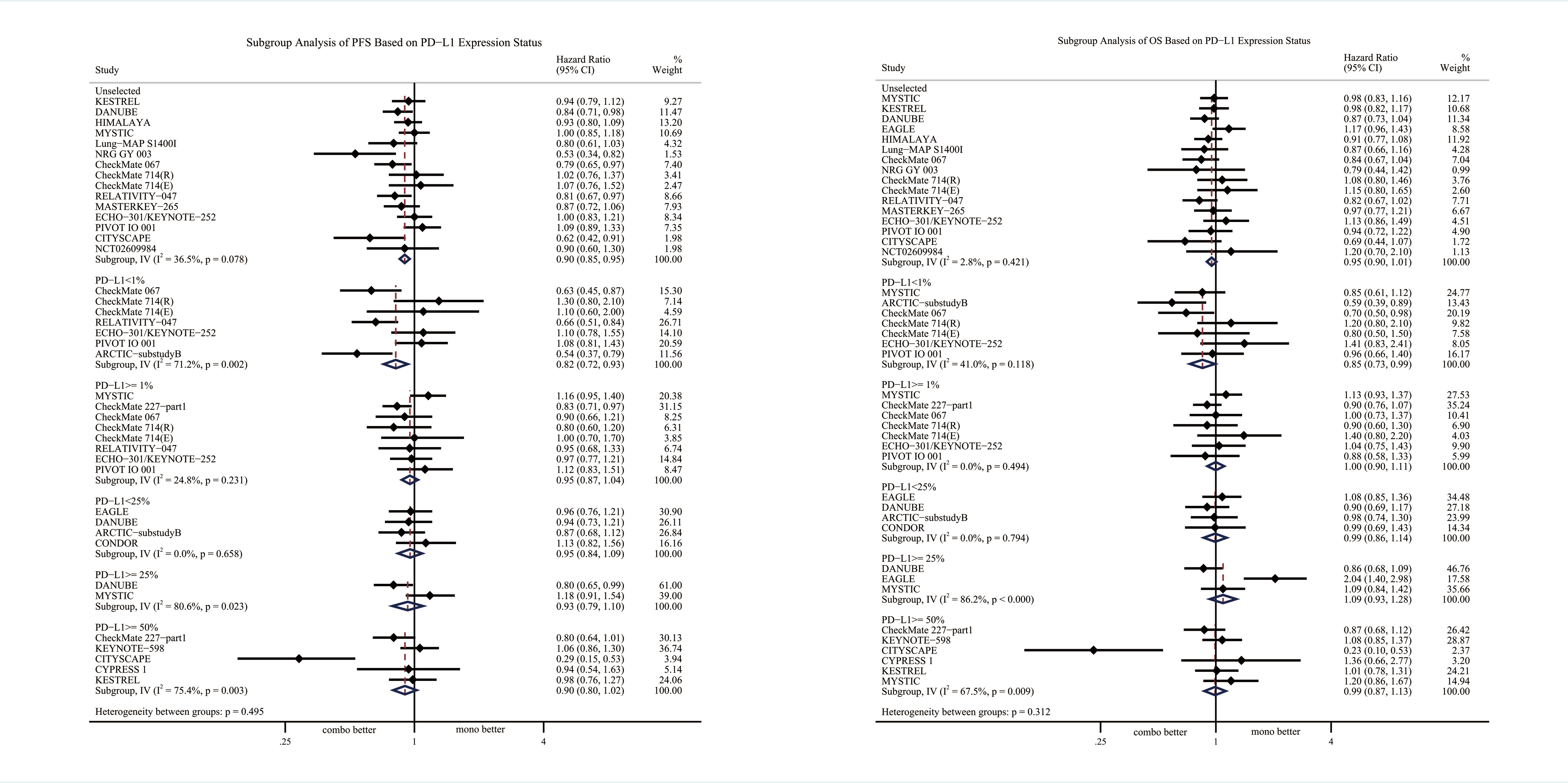


**Supplementary Fig. S4** Forest plots of subgroup analyses of ORR, PFS, OS, any-grade TRAEs and grade higher than or equal to 3 TRAEs based on cancer type. Combo: combination therapy; mono: monotherapy


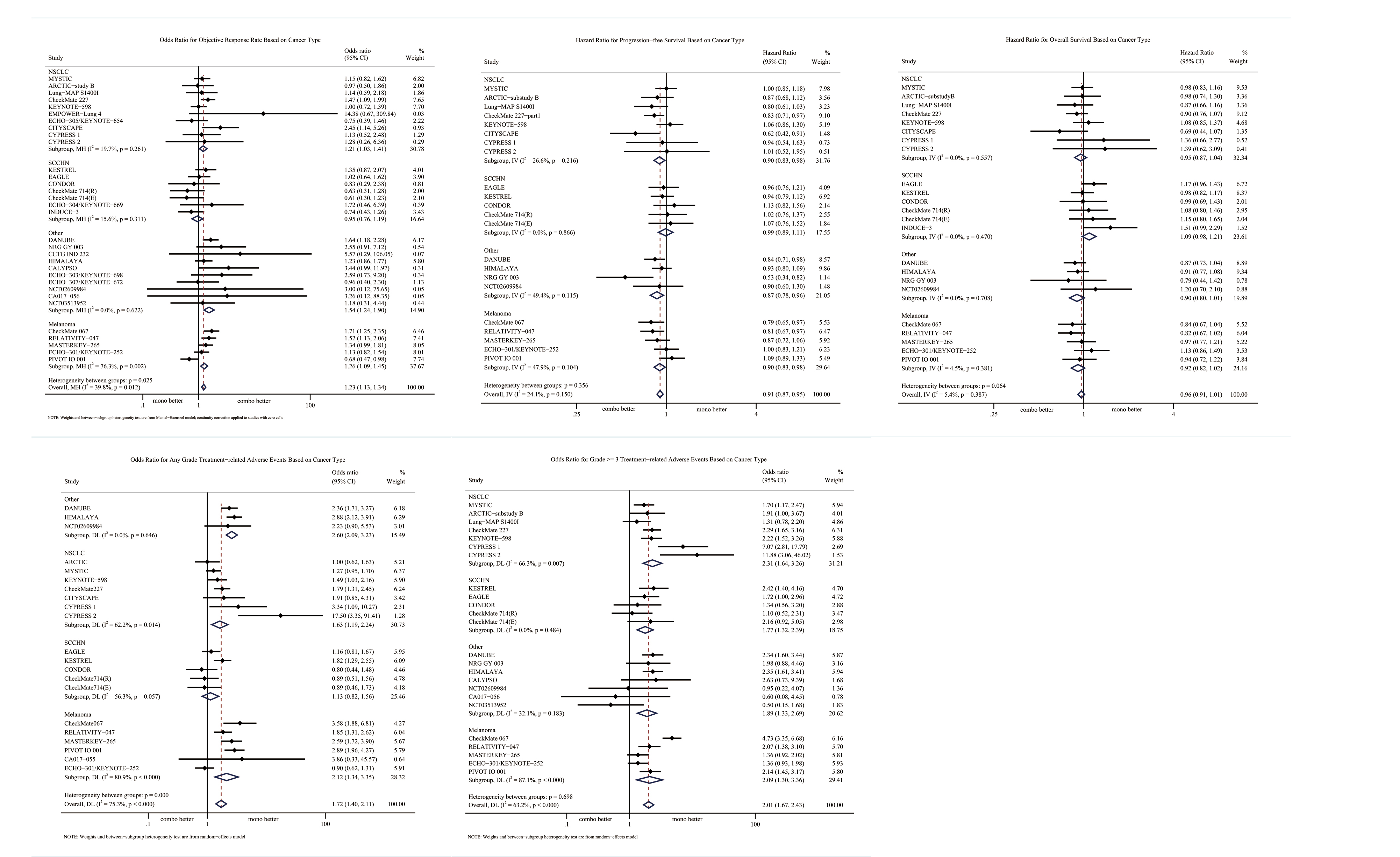


**Supplementary Fig. S5** Forest plots of subgroup analyses of ORR, PFS, OS, any-grade TRAEs and grade higher than or equal to 3 TRAEs based on treatment setting. Combo: combination therapy; mono: monotherapy


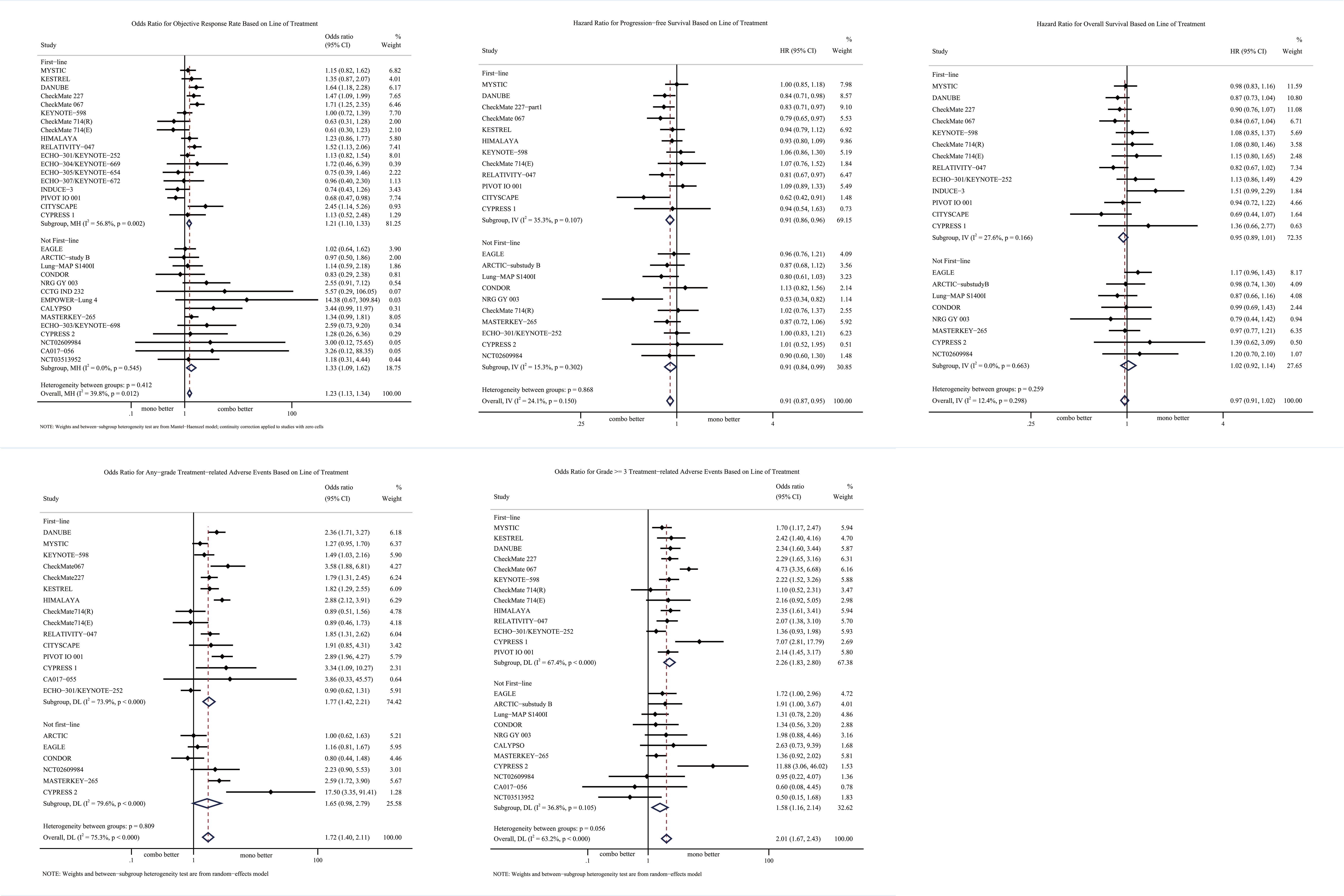


**Supplementary Fig. S6** Funnel plots of ORR, PFS, OS, any-grade TRAEs and grade higher than or equal to 3 TRAEs. Symmetry represents for no significant publication bias.


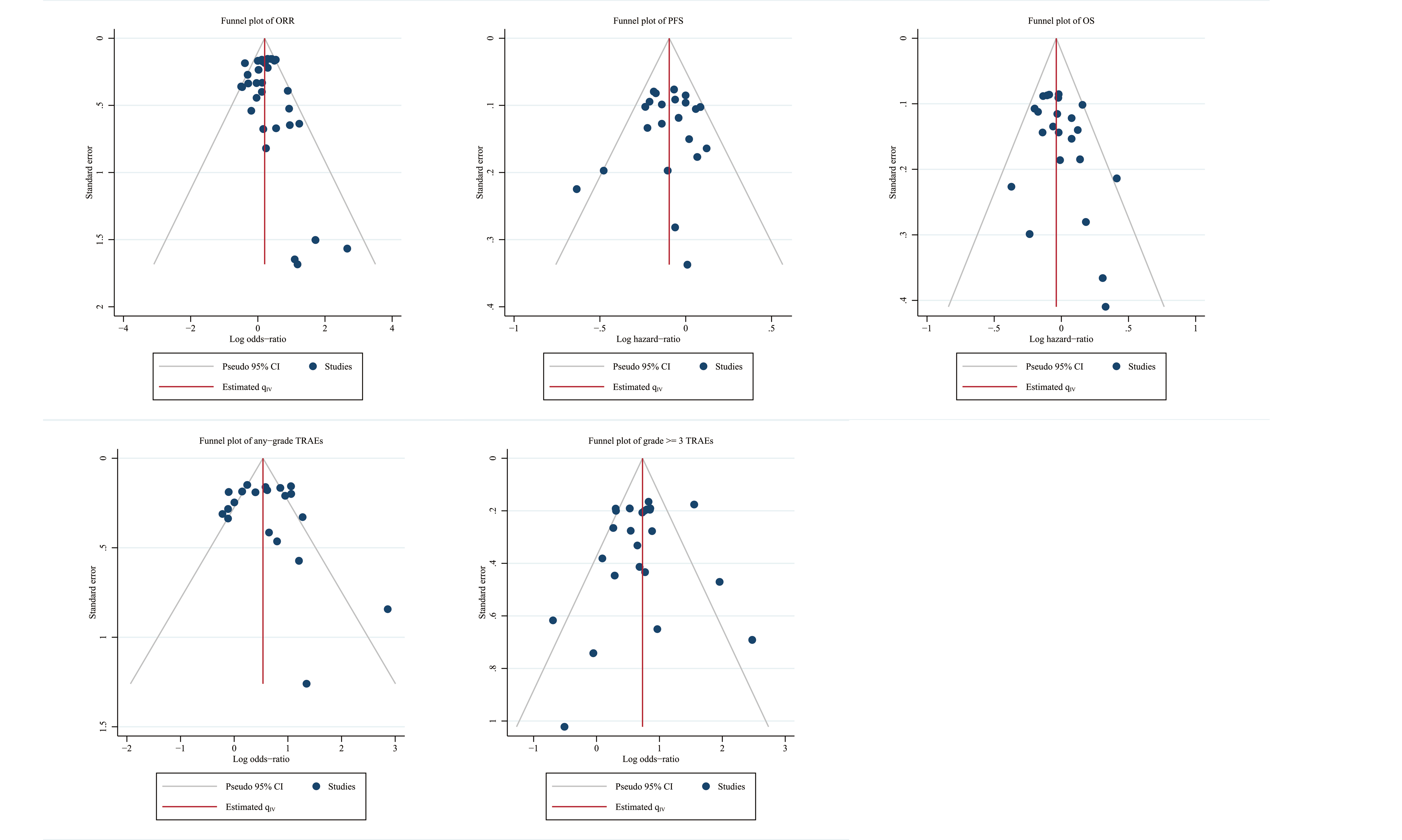

Supplement: Supplementary file 1 — Supplementary file1 (DOCX 9354 KB) [file 262_2024_3734_MOESM1_ESM.docx]
